# Antimicrobial Stewardship Practices of Community Pharmacists in United Arab Emirates

**DOI:** 10.3390/antibiotics12081238

**Published:** 2023-07-27

**Authors:** Mohammad M. AlAhmad, Syed Arman Rabbani, Remah Al-Salman, Hiba Alameri, Yamama Al Namer, Ammar Ali Saleh Jaber

**Affiliations:** 1 Clinical Pharmacy, College of Pharmacy, Al Ain University, Al Ain 64141, United Arab Emirates; 2 AAU Health and Biomedical Research Center, Al Ain University, Abu Dhabi 112612, United Arab Emirates; 3 Department of Clinical Pharmacy and Pharmacology, RAK College of Pharmacy, RAK Medical and Health Sciences University, Ras Al Khaimah 11172, United Arab Emirates; 4 Bausch Health Company, Dubai 23630, United Arab Emirates; 5 Proficiency Healthcare Diagnostics Laboratory, Al Ain 97751, United Arab Emirates; 6 Al Ain Pharmacy Group, Al Ain 1667, United Arab Emirates; 7 Dubai Pharmacy College for Girls, Dubai 19099, United Arab Emirates

**Keywords:** antimicrobial stewardship activities, antibiotics, community pharmacies, community pharmacists

## Abstract

Background: The purpose of this paper is to assess the implementation of antimicrobial stewardship (AMS) activities in community pharmacies in the United Arab Emirates (UAE). Methods: A descriptive cross-sectional study in the Emirate of Abu Dhabi, UAE, was conducted using a validated questionnaire. The questionnaire consisted of four AMS outpatient core elements, namely, commitment, action, tracking and reporting, and education and expertise, with each element containing different associated items. Pharmacy teams’ responses were categorized into three levels: low, satisfactory, or high. Results: Fifty-five pharmacy teams participated. Respondents confirmed implementation of at least one item of each AMS outpatient core element: commitment (94.5%), action (94.5%), tracking and reporting (67.3%), and education and expertise (81.8%). In supporting AMS implementation, surveyed teams scored high (81.8%) for action, satisfactory (65.5%) for education and expertise, low (43.6%) for tracking and reporting, and satisfactory (76.4%) for commitment. Attending antimicrobial stewardship programs was found to be a statistically significant predictor of implementation of antimicrobial stewardship activities (*p* = 0.048). Conclusions: The majority of community pharmacies met the core elements of outpatient antimicrobial stewardship to some degree. There is a significant association between participation in antimicrobial stewardship programs and implementation of antimicrobial stewardship activities by pharmacists in practice.

## 1. Introduction

Antimicrobial resistance (AMR) is a complex and multifaceted global health concern, contributing to millions of deaths worldwide each year, as it jeopardizes antibiotic effectiveness against bacterial infections [1,2]. According to the “Antimicrobial Resistance Collaborators” statistical modes, it was estimated that 4.95 million deaths in 2019 were attributed to bacterial AMR [3].

The irrational use of antimicrobials, both in hospital and community settings, has played a significant role in exacerbating the AMR burden [4]. Furthermore, inadequate public awareness regarding the risks associated with AMR and the lack of comprehensive antimicrobial stewardship (AMS) programs in community settings contribute to the spread of AMR [5].

Community settings, including outpatient clinics and community pharmacies, play a crucial role in the prescription and dispensing of antibiotics [6]. The use of antibiotics in community settings is often marred by practices such as self-medication, inappropriate self-diagnosis, and limited healthcare professional oversight [7]. Community settings account for the majority of antibiotic prescriptions, with approximately 80% of prescriptions being written and dispensed in these settings [8]. Studies have reported that a significant proportion of these prescriptions are inappropriate in terms of choice, dose, duration, or spectrum of antibiotics [9]. This not only raises concerns about patient safety but also contributes to the increasing emergence of AMR in the community.

AMS programs are comprehensive strategies aimed at optimizing the use of antibiotics by the adoption of a multidisciplinary approach constituting policymakers, healthcare providers, researchers, infection control practitioners, and the public. These programs ensure patient safety, improve clinical outcomes, and minimize the emergence and spread of AMR whilst optimizing the therapeutic potential of antibiotics by focusing on evidence-based practices. Such activities include reviewing antimicrobial utilization, educating healthcare providers and providing them with feedback on their performance, examining antimicrobial use, and monitoring resistance patterns [10].

While AMS programs have traditionally been implemented in hospital settings, the expansion of such programs to community settings is critical. Community settings offer an opportunity to promote responsible antibiotic use and educate patients on AMR. Establishing AMS programs in community settings can help address the challenges associated with inappropriate antibiotic use and contribute to the containment of AMR [11].

Community pharmacists play a crucial role in the healthcare system, particularly in the context of AMS in community settings [12]. They are often the first point of contact for patients seeking medication and can provide valuable guidance on the appropriate use of antibiotics. However, studies have shown variations in the AMS practices of community pharmacists, including knowledge gaps, inadequate counseling, and inconsistent adherence to guidelines [13,14]. Understanding the current practices of community pharmacists in relation to AMS is crucial for identifying areas for improvement and developing targeted interventions to enhance their role in promoting responsible antibiotic use.

Furthermore, community pharmacists are well-positioned to contribute significantly to the implementation of AMS activities in community settings [15]. They can actively engage in patient education, promote rational antibiotic use, and provide appropriate counseling on medication adherence and potential adverse effects [16]. Additionally, community pharmacists can collaborate with other healthcare professionals, such as physicians and nurses, to ensure a multidisciplinary approach to the implementation of AMS programs [17]. The core elements of a successful AMS program in the outpatient setting were outlined by the US Centers for Disease Control (CDC). They include a commitment to maximizing patient safety and optimizing the prescription of antibiotics, implementing policy and practice, monitoring the prescription of antibiotics and reporting feedback to clinicians, and educating physicians and patients on the prescribing actions of antibiotics [18].

In the United Arab Emirates (UAE), the Abu Dhabi Department of Health (DOH) has produced several health regulations and guidelines for AMS programs and practices. Examples of such initiatives include the Abu Dhabi Antimicrobial Stewardship Program, the Dubai Antimicrobial Stewardship Taskforce, the Sheikh Khalifa Medical City Antimicrobial Stewardship Program, the Cleveland Clinic Abu Dhabi Antimicrobial Stewardship Program (CCAD ASP), the Rashid Hospital Antimicrobial Stewardship (RH AMS) Program, and the Tawam Hospital Antimicrobial Stewardship Program [19].

Studies by Abduelkarem AR et al. (2022) and Jairoun S. (2023) showed the high prevalence of the self-medication of antibiotics without prescriptions in the population and emphasized the need to improve public awareness and encourage UAE pharmacists to participate in special programs to optimize the rational use of antimicrobials [20,21]. Another study examined hospital pharmacists’ knowledge and confidence regarding antimicrobial stewardship and concluded that hospital pharmacists in the UAE have good knowledge and a high level of confidence in implementing and integrating AMS initiatives in the UAE [22].

Given this background, and to the best of our knowledge, the extent to which community pharmacists in the UAE implement AMS activities in their daily practice is unknown. Therefore, this is the first study that aims to assess the AMS practices of community pharmacists in the UAE. By examining these practices, this study aims to provide a comprehensive understanding of the current state of AMS in UAE community pharmacies.

## 2. Results

A detailed description of the socio-demographic data of the participants is presented in Table 1. The response rate was 100%; thus, responses from *n* = 55 participants (pharmacy teams) were subjected to further analysis. In total, 58.2% (*n*= 32) of the respondents fell within the age group of 25–35 years, while 41.8% (*n* = 23) were aged between 35 and 45 years. The majority of the pharmacy teams was comprised of both males and females (90.9%), while 9.1% (*n* = 5) were composed of only females. With regards to the location of the community pharmacy, 47.3% (*n* = 26) were situated in urban areas, 36.4% (*n* = 20) were attached to medical centers, and 16.4% [9] were located in malls. Furthermore, 76.4% (*n* = 42) of the pharmacy teams had bachelor’s degrees, while 10.9% and 12.7% had bachelor’s degrees with certificates and bachelor’s with master’s degrees, respectively. In terms of experience, 65.5% (*n* = 36) had an average of 5–10 years of team experience, while 27.3% and 7.3% of the pharmacy teams had <5 years and >10 years of experience, respectively. The majority of pharmacy teams (80%) were employed by large chain pharmacies (more than 20); 63.6% (*n* = 35) of the participants achieved less than 100 prescriptions per day; and 76.4% had not attended antimicrobial stewardship programs, conferences, or CMEs (continuing medical education).

The four outpatient core elements and the items proposed for them were assessed with yes/no questions. Accordingly, the antimicrobial stewardship practices in the community pharmacies that met the core elements of the outpatients were assessed. The outpatient core elements included commitment, action, tracking and reporting, and education and expertise. Regarding commitment, the rate of affirmative responses to the ability of the facility to demonstrate commitment and responsibility for optimizing antibiotic prescribing was 94.5%. As shown in Table 2, 80% of respondents answered in the affirmative to the question of whether the public is consistently informed about the appropriate use of antimicrobials. On the other hand, none of the respondents (0%) had specific job descriptions and adequate periodic evaluation of the pharmacist’s antimicrobial stewardship responsibilities. On average, less than 26% displayed posters and fliers regarding antibiotic stewardship, identified a single leader in direct antibiotic stewardship, joined a local stewardship to work with prescribers, and pursued certification in antimicrobial stewardship.

In terms of action, 94.5% of respondents confirmed that the facility where they work has at least one policy or practice in place to improve antibiotic prescribing; 52.7% ensured that healthcare providers fully completed prescribing elements, particularly weighting for pediatric prescriptions to allow for verification of doses; 60% ensured that healthcare providers included prescription durations of selected antimicrobials in the healthcare system used to enable proper and complete reviews of selected antimicrobials (i.e., agents, doses, frequency, and duration); 57.2% appropriately assessed patient signs and symptoms and considered over-the-counter medications (OTCs) to improve patient management; while 47.3% called and clarified prescriptions for discrepancies with diagnoses or ICD-10 and standards. As shown in Table 2, response rates for the other items were less than 33%.

Regarding tracking and reporting, 67.3% confirmed that the facility monitors at least one aspect of antibiotic prescribing. For underlying aspects, 23.6% of respondents tracked and reported improvements in specific patients based on facility policies and regulations; 27.3% recalled individuals who had received antibiotics 24 to 48 h earlier, tracked and reported antibiotic complications, and encouraged them to contact their provider if needed, while 36.4% reported that the process of reviewing prescriptions, including the agent, dose, frequency, and duration, was accurate. The other measures under this core element were implemented at a rate of <20%.

The fourth core element is education and expertise, which consists of several items in two categories: Patient Education on Antimicrobial Stewardship, and Clinician Education on Antimicrobial Stewardship. In total, 81.8% confirmed that the facility provides clinicians and patients with resources on evidence-based antibiotic prescribing; 76.4% educated patients, and 54.5% educated clinicians about antimicrobial stewardship activities; 60% used effective communication strategies to educate patients about the appropriate need for antibiotics; 60% educated patients about the potential harms of antibiotic treatment; and 61.8% educated patients about the serious side effects of antibiotics. In addition, 67.3% provided continuing education for pharmacists and technicians.

As described earlier, the surveyed teams were ranked based on their responses in supporting the implementation of antimicrobial stewardship activities in community pharmacies for each outpatient core element. The responses of the pharmacy teams were classified into three categories: low (if they answered “no” for the respective outpatient core element), satisfactory (if they answered “yes” for one or two items of the respective outpatient core element), or high (if they answered “yes” for more than two items of the respective element). As observed in Table 3, the proportion of teams that answered “no” (i.e., low rank) was significantly low for commitment (5.5%) and action (5.5%). Higher percentages of the low rank were recorded for education and expertise (20%) and tracking and reporting (32%). Conversely, “action” recorded the highest percentage for teams that answered “yes” for more than two items of the respective element (i.e., high rank), with commitment showing the lowest percentage for the high rank at 18.2%. In addition, the highest percentage for the satisfactory rank was reported for commitment at 76.4%, while action had the lowest percentage at this rank.

To predict the implementation of antimicrobial stewardship activities in community pharmacies, we assumed that community pharmacies strongly supported the implementation of antimicrobial stewardship activities if they performed highly in at least three outpatient core elements. A contingency table analysis and chi-square post hoc testing were used to test the association between community pharmacies’ performance regarding antimicrobial stewardship implementation. The value of the z test at a 95% level of significance was ±1.96.

Evidently, as shown in Table 4, only attending antimicrobial stewardship programs, conferences, or CMEs was found to be a statistically significant predictor of the implementation of antimicrobial stewardship activities at a *p*-value of 0.048 (*X*^2^ = 8.69 (4)). For the rest of the variables with *p*-values greater than 0.05, it could be deduced that the null hypothesis could not be rejected, implying there was not enough evidence to indicate an association between the variables and implementation of antimicrobial stewardship activities by the community pharmacies.

## 3. Discussion

Given the apparent severe health outcomes related to infections caused by bacteria and the curb of novel therapeutic interventions, antibiotic resistance has evolved into a global health concern [23]. Since their initiation, antimicrobial stewardship initiatives have shown exceeding effectiveness in lowering the rate of antibiotic misuse [24]. However, there is a dearth of studies investigating the role of community pharmacists in antimicrobial stewardship in the study location. Thus, elucidating the current trends and predictors of the effective implementation of antimicrobial stewardship activities in community pharmacies is critical to optimizing the role of community pharmacists as antimicrobial stewards [25]. This study demonstrates that current practices in community pharmacies vary widely in implementing the core elements of antimicrobial stewardship in the outpatient setting, and that few of them (36.4%) successfully implement the measures recommended in each core element.

In this study, the majority of community pharmacy teams was aged between 25 and 35; was comprised of males and females; had only bachelor’s degrees; had experience of between 5 and 10 years; worked in large chain pharmacies located in urban areas; gave out less than 100 prescriptions/day; and did not attend antimicrobial stewardship programs, conferences, or CMEs. In contrast, 74% of all eligible pharmacies in England actively participate in structured antimicrobial stewardship programs [26]. This high rate of participation can be attributed to the fact that England has the English Pharmacy Quality Scheme (PQS), which has included a domain for antimicrobial stewardship with the aim of embedding and building upon antimicrobial stewardship activities over time. To ensure clinical effectiveness, patient safety, and patient experience in the use of antibiotics, a similar program needs to be incorporated.

The measure of core elements of outpatient antimicrobial stewardship is a vital approach to precisely describing the impact of antimicrobial stewardship practices. Given the different stewardship opportunities for pharmacists, certain stewardship strategies may be more easily implemented compared to others, depending on the practice setting. Thus, this study explored how the implementation of antimicrobial stewardship activities in community pharmacies varied with each outpatient core element. The core elements of implementation of the antimicrobial stewardship activities in the community were ranked from low to satisfactory to high. Action and education and expertise were ranked relatively high, commitment was mostly ranked satisfactory, and tracking and reporting was distributed across the ranks. It can be inferred that the majority of community pharmacies met the core elements of outpatient antimicrobial stewardship. This is quite phenomenal, as a study conducted in the US reported that most pharmacies do not meet the core elements [25]. This disparity can be attributed to the fact that some strategies might be more easily implemented than others. For instance, this study reported a high rate for the use of consistent messages by staff to communicate with the public about antibiotics under the commitment element. On the other hand, the American Society of Health System Pharmacists suggested the provision of antibiotic education and counseling to patients and the participation in public health programs to stem the spread of infections [27]. Both stewardship strategies can be easily implemented since community pharmacists are already actively involved in public health care schemes and are key contributors to immunization programs, patient education, and vaccine administration.

The lower rates for the other strategies under the various core elements can be attributed to challenges faced in their implementation [28]; thus, there is a need for additional training of pharmacists to expand their roles and capabilities in outpatient antimicrobial stewardship activities. Nkinda L. et al., (2022) reported a dearth of laboratory facilities to support culture and susceptibility tests, the absence of materials and reagents, pressure from management to avoid loss or to generate income, interference from patients, and inadequate training opportunities as barriers to the implementation of the stewardship activities [29].

Furthermore, this study found a significant association between attending antimicrobial stewardship programs, conferences, or CMEs and the implementation of antimicrobial stewardship activities by community pharmacists. This suggests that the poor implementation of some of the stewardship strategies is due to the inability of community pharmacists to update their stewardship knowledge on effective implementation. Therefore, community pharmacists require additional training by attending relevant programs to enhance their stewardship capabilities.

The generalizability of the research findings may be limited by a number of constraints. Although this research was conducted in the largest emirate in the UAE, the results cannot be generalized to all CPs in the country. Second, the data collected could be biased due to the disparity among respondent accuracy, whether the entirety of the questionnaire was completed, and the under- or over-reporting of variables related to AMS activities. Nonetheless, our results provide rationalized insight into community-based antimicrobial stewardship activities.

## 4. Materials and Methods

### 4.1. Study Design and Setting

This descriptive cross-sectional study followed the STROBE statement for reporting observational cross-sectional studies and was conducted in the community pharmacies of Abu Dhabi. Abu Dhabi is the capital city and the largest emirate in the United Arab Emirates (UAE). It is located on the southeastern side of the Arabian Peninsula, on the coast of the Arabian Gulf, and has a population of 1,540,000.

### 4.2. Study Population and Sample Size

We employed stratified random sampling to ensure representation from all the geographic regions of the Emirate of Abu Dhabi. We divided Abu Dhabi into six main geographical areas, and all community pharmacies in each area were screened in accordance with the study criteria. Community pharmacies having pharmacy teams with at least four pharmacists were considered for inclusion in the study. We selected a sample of four or more community pharmacies from each area, with the final number being dependent on the cluster of pharmacies in a given area and the willingness of pharmacy teams to participate in the study. This approach ensured that our sample was geographically representative of all community pharmacies in Abu Dhabi. This disproportionate stratified random sampling resulted in 55 community pharmacies (pharmacy teams) being included in the study.

### 4.3. Study Instrument

The study instrument was developed using a systematic approach. The initial step involved reviewing published literature on AMS in community settings. We used the Centers for Disease Control and Prevention’s (CDC) core elements of outpatient antimicrobial stewardship [18,30] and AMS surveys related to practices of community pharmacists reported in the literature [31,32,33,34] for developing the study instrument. Following initial development, the study instrument was reviewed for content validity by a panel of experts consisting of researchers in the field of AMS and pharmacy practice, as well as community pharmacists. All recommended modifications were conducted as per the experts’ feedback. After the expert review, the instrument underwent a pilot testing phase. The study instrument was pre-tested with 10 community pharmacists. The pilot testing of the instrument yielded a Cronbach’s alpha of 0.868, demonstrating high reliability.

The study instrument consisted of four core elements related to outpatient AMS activities, namely, Commitment, Action, Tracking and Reporting, and Education and Expertise, with each of the elements having different related items. These items were evaluated using yes or no responses. Depending on the responses, the community pharmacy teams were categorized into three groups: low (if they responded “no” to all the items of a respective core element), satisfactory (if they responded “yes” to 1 or 2 items of a respective core element), or high (if they responded “yes” to more than 2 items of a core element).

### 4.4. Data Collection and Analysis

The study instrument was distributed by the study investigators to the selected community pharmacies in the Abu Dhabi region. Written informed consent was obtained from the participants before administration of the study instrument. The study investigators clarified doubts of the participants and addressed the questions that arose during the self-administration of the questionnaire (Supplementary Materials).

Statistical Package for the Social Sciences (SPSS) version 27.0 was used for data analysis. The skewness and kurtosis of the data were evaluated before analysis. The Shapiro–Wilk test was utilized to verify the data’s normality. Categorical variables were described using frequency and percentage with 95% confidence intervals. Contingency table analysis and chi-square post hoc testing were used to test the association between the community pharmacies’ performance regarding AMS practices. The value of the z test at a 95% level of significance was ±1.96.

### 4.5. Ethics Approval

The study was conducted in compliance with the European Union General Data Protection Regulation (GDPR) and met Al Ain University’s requirements for confidentiality, data use, and participant protection through anonymous data collection and storage. Ethics approval for the study was granted by the Research Ethics Committee of Al Ain University (reference number COP/AREC/AD/21).

## 5. Conclusions

It can be inferred that the majority of community pharmacies met the core elements of outpatient antimicrobial stewardship to a certain extent, with action and education and expertise core elements ranking relatively high in the implementation of antimicrobial stewardship activities. The lower rates for some of the strategies under the various core elements can be attributed to challenges faced in their implementation. This study found a significant association between attending antimicrobial stewardship programs, conferences, or CMEs and the implementation of antimicrobial stewardship activities by community pharmacists, which implies that the poor implementation of some of the stewardship strategies is due to the inability of the community pharmacists to improve upon their stewardship knowledge and practices.

## Figures and Tables

**Table 1:** Socio-demographic characteristics of community pharmacy teams (*n* = 55).

Socio-Demographic Characteristics	*n* (%)	95% CI
Average team age (years)		
25–35	32 (58.2)	44.1–71.3
35–45	23 (41.8)	28.7–55.9
Team genders		
Females	5 (9.1)	3–20
Males and females	50 (90.9)	80–97
Education level		
Bachelor’s	42 (76.4)	63–86.8
Bachelor’s and certificates	6 (10.9)	4.1–22.2
Bachelor’s and master’s	7 (12.7)	5.3–24.5
Average team experience		
Less than 5 years	4 (7.3)	2–17.6
5–10 years	36 (65.5)	51.4–77.8
More than 10 years	15 (27.3)	16.1–41
Pharmacy location		
Mall	9 (16.4)	7.8–28.8
Attached to medical center	20 (36.4)	23.8–50.4
Urban area	26 (47.3)	33.7–61.2
Type of pharmacy		
Independent pharmacy	4 (7.3)	2–17.6
Small chain pharmacy (less than 20)	7 (12.7)	5.3–24.5
Large chain pharmacy (more than 20)	44 (80)	67–89.6
Number of prescriptions/day		
Less than 100 prescriptions/day	35 (63.6)	49.6–76.2
100–300 prescriptions/day	14 (25.5)	14.7–39
More than 300 prescriptions/day	6 (10.9)	4.1–22.2
Attending antimicrobial stewardship programs, conferences, or CMEs		
None	42 (76.4)	63–86.8
1–2 pharmacists	10 (18.2)	9.1–30.9
More than 2 pharmacists	3 (5.5)	1.1–15.1

*n* (%) = number of participants (percentage). CME: continuing medical education.

**Table 2:** Four outpatient core elements and their proposed items.

Core Elements and Their Proposed Items	*n* (%)	95% CI
Core element no. 1: Commitment		
Does the facility demonstrate commitment and responsibility for optimizing antibiotic prescribing and patient safety related to antibiotics? If yes, please indicate which of the following actions have been taken; select all that apply.	52 (94.5)	84.9–98.9
● Post special forms and posters to provide information on the use of antimicrobials	14 (25.5)	14.7–39
● Assign a qualified pharmacist as team leader to manage antimicrobial stewardship activities in a facility.	13 (23.6)	13.2–37
● The facility provides specific job descriptions and appropriate periodic evaluation of the pharmacist’s antimicrobial stewardship responsibilities	0 (0)	0–6.5
● The pharmacy team consistently communicates messages to the public about appropriate antimicrobial use.	44 (80)	67–89.6
● Facilitate collaboration with prescribing physicians and other health care professionals to improve antimicrobial use.	13 (23.6)	13.2–37
● A member of the pharmacy team completes specialized courses and is successfully certified in infectious disease and antimicrobial stewardship.	14 (25.5)	14.7–39
Core element no. 2: Action		
Has the facility implemented at least one policy or practice to improve antibiotic prescribing? If yes, please indicate which of the following actions have been taken; select all that apply.	52 (94.5)	84.9–98.9
● Ensure healthcare providers include duration of selected antimicrobials in the healthcare system used to allow proper and complete review of selected antimicrobials (i.e., agents, doses, frequency, duration).	33 (60)	45.9–73
● Ensure healthcare providers fully complete prescription elements, especially weighting for pediatric prescriptions, to allow for verification of doses.	29 (52.7)	38.8–66.3
● Offer appropriate alternatives, especially if treatment is not evidence-based.	6 (10.9)	4.1–22.2
● Notify the healthcare provider if the antibiotic selected appears to be redundant in terms of coverage of the spectrum of activity.	10 (18.2)	9.1–30.9
● Review the correct dose to optimize antimicrobial dosing.	16 (29.1)	17.6–42.9
● Review the correct treatment duration to avoid overprescribing.	16 (29.1)	17.6–42.9
● Review the antimicrobial medication re-prescribing (refills) based on indications.	17 (30.9)	19.1–44.8
● Patients found to be allergic to penicillin without prior testing or with a history of allergy inconsistent with a true allergy are to be referred for further evaluation and confirmation.	18 (32.7)	20.7–46.7
● Appropriately assess patients’ signs and symptoms, taking into account over-the-counter medications (OTCs), to improve patient management.	32 (57.2)	44.1–71.3
● Improve the pharmacist’s ability to address the benefits and harms of antibiotic treatment through communication skills training.	16 (29.1)	17.6–42.9
● Appropriately assess the patient’s condition when treating self-limiting diseases.	20 (36.4)	23.8–50.4
● Assist the clinician in managing patient expectations for antibiotics.	10 (18.2)	9.1–30.9
● The callback script is effectively used to clarify and recommend the selected antimicrobial, dose, frequency, duration, de-escalation, discontinuation, etc.	15 (27.3)	16.1–41
● Call and clarify prescriptions when there are discrepancies with diagnoses or ICD-10 and standards.	26 (47.3)	33.7–61.2
● Provide patients with printed instructions with recommendations for OTC medications that might help improve their symptoms.	0 (0)	0–6.5
● Use a standard approach of recommendations to help patients when OTC medications are a valid option to control their symptoms.	10 (18.2)	9.1–30.9
● Consult with patients to determine their needs and agree on a set of recommendations to relieve their symptoms.	16 (29.1)	17.6–42.9
● Provide instructions on when to contact the healthcare provider if complications or side effects occur or the medication is not working for the patient.	16 (29.1)	17.6–42.9
● Provide immunizations for the patient based on those available at the pharmacy.	11 (20)	10.4–33
● Review the patient’s immunization status and discuss recommended immunizations, including influenza vaccination during flu season.	8 (14.5)	6.5–26.7
Core element no. 3: Tracking and Reporting		
Does the facility monitor at least one aspect of antibiotic prescribing? If yes, please indicate which of the following measures have been taken; select all that apply.	37 (67.3)	53.3–79.3
● Track and report improvements in specific patients based on facility policies and regulations	13 (23.6)	13.2–37
● Regularly monitor antibiotic prescribing and provide feedback to clinicians on their antimicrobial prescribing practices.	10 (18.2)	9.1–30.9
● Track and monitor antibiotic use for respiratory infections, as these are the most commonly used antibiotics and contribute greatly to antibiotic misuse or unnecessary use.	8 (14.5)	6.5–26.7
● Report and track healthcare providers’ acceptance of pharmacists’ recommendations for antibiotic use. Share feedback and evidence-based recommendations.	5 (9.1)	3–20
● Regularly track and report on the percentage of antibiotic use and dispensing. Alert local authorities when agreed-upon usage levels are exceeded.	7 (12.7)	5.3–24.5
● Create a percentage analysis of all dispensed medications, including antibiotics, to determine if there are prescribers who are driving up the numbers and alert them to their consumption.	11 (20)	10.4–33
● Create a percentage analysis of specific antibiotics that need special attention (reserved for more severe cases) based on antibiotic classification (first, second, or third choice) to determine if there are prescribers driving up the numbers and make them aware of their consumption.	9 (16.4)	7.8–28.8
● Recall individuals who received antibiotics 24 to 48 h earlier. Track and report antibiotic complications and ask them to contact their provider if needed.	15 (27.3)	16.1–41
● Report the process of reviewing orders, namely, if the agent, dose, frequency, and duration were correct.	20 (36.4)	23.8–50.4
● Track and determine the percentage of individuals who were educated as measured by the number of individuals who received antibiotics.	9 (16.4)	7.8–28.8
Core element no. 4: Education and Expertise		
Does the facility provide resources to clinicians and patients on evidence-based antibiotic prescribing? If yes, please indicate which of the following actions have been taken; select all that apply.	45 (81.8)	69.1–90.9
Antimicrobial stewardship education to patients	42 (76.4)	63–86.8
● Educate patients about when antimicrobials are and are not needed using appropriate communication techniques (e.g., how to control symptoms when antimicrobials are not needed, and when to contact healthcare providers if the case does not improve or worsens).	33 (60)	45.9–73
● Educate patients about the potential harms and complications of antimicrobial use.	33 (60)	45.9–73
● Educating patients about antimicrobial side effects such as superinfection, diarrhea, *Clostridium difficile* infection, and others such as allergic reactions.	34 (61.8)	47.7–74.6
● Education can be oral or written. Inquire if the patient has any questions.	20 (36.4)	23.8–50.4
● Provide information about preventive medicine and wellness initiatives (e.g., immunizations).	15 (27.3)	16.1–41
● Provide patient education in various forms (e.g., posters, brochures, Facebook, website, public presentations in the community).	16 (29.1)	17.6–42.9
● Ensure health literacy.	20 (36.4)	23.8–50.4
Antimicrobial stewardship education to clinicians	30 (54.5)	40.6–68
● Provide in-person training (e.g., reinforcement techniques when training is not delivered as expected, inclusion of antimicrobial control training in new employee orientation).	16 (29.1)	17.6–42.9
● Provide continuing education for pharmacists and technicians.	37 (67.3)	53.3–79.3
● Ensure timely access to individuals with expertise.	21 (38.2)	25.4–52.3

*n* (%) = number of participants (percentage).

**Table 3:** Ranking of the surveyed teams by their responses in supporting the implementation of antimicrobial stewardship activities in community pharmacies in each outpatient core element.

Outpatient Core Elements	Low *n* (%)	Satisfactory *n* (%)	High *n* (%)
Commitment	3 (5.5%)	42 (76.4%)	10 (18.2%)
Action	3 (5.5%)	7 (12.7%)	45 (81.8%)
Tracking and reporting	18 (32.7%)	13 (23.6%)	24 (43.6%)
Education and expertise	11 (20%)	8 (14.5%)	36 (65.5%)

*n* (%) = number of participants (percentage).

**Table 4:** Predictors of antimicrobial stewardship activities implementation in community pharmacies.

Variable	Community Pharmacy Supported the Implementation of Antimicrobial Stewardship Activities: Contingency Table Analysis and Chi-Square Post Hoc Testing				
	Low *n*; % (z-Test Score)	Satisfactory *n*; % (z-Test Score)	High *n*; % (z-Test Score)	Pearson Chi-Square Value (df)	*p*-Value ^1^
Average team age					
25–35	10; 31.3% (0.8)	14; 43.8% (1.3)	8; 25% (−2.1)	4.3 (2)	0.116
35–45	5; 21.7% (−0.8)	6; 26.1% (−1.3)	12; 52.2% (2.1)		
Team genders					
Females	0; 0% (−1.4)	2; 40% (0.2)	3; 60% (1.2)	2.36 (2)	0.165
Males and females	15; 30% (1.4)	18; 36% (−0.2)	17; 34% (−1.2)		
Education level					
Bachelor’s	12; 28.6% (0.4)	17; 40.5% (1.1)	13; 31% (−1.5)	2.97 (4)	0.544
Bachelor’s and certificates	2; 33.3% (0.4)	1; 16.7% (−1.1)	3; 50% (0.7)		
Bachelor’s and master’s	1; 14.3% (−0.8)	2; 28.6% (−0.5)	4; 57.1% (1.2)		
Average team experience					
Less than 5 years	1; 25% (−0.1)	2; 50% (0.6)	1; 25% (−0.5)	2.76 (4)	0.611
5–10 years	11; 30.6% (0.8)	14; 38.9% (0.5)	11; 30.6% (−1.2)		
More than 10 years	3; 20% (−0.7)	4; 26.7% (−0.9)	8; 53.3% (1.6)		
Pharmacy location					
Mall	4; 44.4% (1.3)	4; 44.4% (0.6)	1; 11.1% (−1.7)	7.58 (4)	0.077
Attached to medical center	2; 10% (−2.2)	7; 35% (−0.2)	11; 55% (2.2)		
Urban area	9; 34.6% (1.2)	9; 34.6% (−0.3)	8; 30.8% (−0.8)		
Type of pharmacy					
Independent pharmacy	2; 50% (1.1)	0; 0% (−1.6)	2; 50% (0.6)	4.52 (4)	0.199
Small chain pharmacy (less than 20)	3; 42.9% (1)	3; 42.9% (0.4)	1; 14.3% (−1.3)		
Large chain pharmacy (more than 20)	10; 22.7% (−1.5)	17; 38.6% (0.7)	17; 38.6% (0.7)		
Number of prescriptions/day					
Less than 100 prescriptions/day	11; 31.4% (0.9)	13; 37.1% (0.2)	11; 31.4% (−1)	7.66 (4)	0.065
100–300 prescriptions/day	3; 21.4% (−0.6)	7; 50% (1.2)	4; 28.6% (−0.7)		
More than 300 prescriptions/day	1; 16.7% (−0.6)	0; 0% (−2)	5; 83.3% (2.5)		
Attending antimicrobial stewardship programs, conferences, or CMEs					
None	13; 31% (1.1)	18; 42.9% (1.8)	11; 26.2% (−2.8)	8.69 (4)	0.048
1–2 pharmacists	2; 20% (−0.6)	1; 10% (−1.9)	7; 70% (2.4)		
More than 2 pharmacists	0; 0% (−1.1)	1; 33.3% (−0.1)	2; 66.7% (1.1)		

^1^ *p* < 0.005 was considered significant. The value of z at a 95% level of significance is ±1.96. *n*; % = number of participants; percentage.

## Data Availability

Not applicable.

## Supplementary Materials

The following supporting information can be downloaded at: https://www.mdpi.com/article/10.3390/antibiotics12081238/s1.
